# Unlocking the power of reflection: how simulation debriefing shapes clinical decision-making and competence in nursing students

**DOI:** 10.3389/fmed.2026.1896558

**Published:** 2026-10-01

**Authors:** Shaimaa Mohamed Amin, Sameer A. Alkubati, Haitham Mokhtar Mohamed Abdallah, Radwa Ahmed Abdel Razek Emara, Nagwa Yehya Ahmed Sabrah, Ibrahim Alasqah, Mona Metwally El-Sayed, Sally Mohammed Farghaly Abdelaliem, Amany Anwar Saeed Alabdullah, Ahmed Abdelwahab Ibrahim El-Sayed

**Affiliations:** 1 Department of Community, Psychiatric, and Mental Health Nursing, College of Nursing, Qassim University, Buraydah, Saudi Arabia; 2 Community Health Nursing Department, Faculty of Nursing, Damanhour University, Damanhour, Egypt; 3 Nursing Services Department, Qassim University Medical City, Buraydah, Saudi Arabia; 4 Department of Medical Surgical Nursing, College of Nursing, University of Ha'il, Ha'il, Saudi Arabia; 5 Medical Surgical Nursing Department, College of Nursing, Jouf University, Sakaka, Saudi Arabia; 6 Faculty of Nursing, Zagazig University, Zagazig, Egypt; 7 Psychiatric and Mental Health Nursing Department, RAK College of Nursing, RAK Medical and Health Sciences University, Ras Al Khaimah, United Arab Emirates; 8 Psychiatric and Mental Health Nursing Department, Faculty of Nursing, Zagazig University, Zagazig, Egypt; 9 Critical Care and Emergency Nursing Department, Faculty of Nursing, Mansoura University, Mansoura, Egypt; 10 School of Health, University of New United Kingdom, Armidale, NSW, Australia; 11 Department of Nursing, Faculty of Allied Health Sciences, Kuwait University, Kuwait City, Kuwait; 12 Department of Nursing Administration, Faculty of Nursing, Alexandria University, Alexandria, Egypt; 13 Department of Maternity and Pediatric Nursing, College of Nursing, Princess Nourah bint Abdulrahman University, Riyadh, Saudi Arabia; 14 Nursing Administration Department, Faculty of Nursing, Alexandria University, Alexandria, Egypt

**Keywords:** clinical decision-making, experiential learning, mediating effect model, nursing competence, simulation, simulation-based education, undergraduate nursing students

## Abstract

**Background:**

Simulation debriefing is widely recognized as a critical component of simulation-based education, yet its specific contribution to clinical decision-making and competence development among undergraduate nursing students remains insufficiently explored.

**Objective:**

To examine the impact of simulation debriefing on clinical decision-making and nursing competence among nursing students and to determine whether clinical decision-making mediates this relationship.

**Methods:**

A multi-center, cross-sectional study was conducted among 650 third- and fourth-year undergraduate nursing students from two XX universities. Participants completed validated instruments assessing debriefing experiences, clinical decision-making, and nursing competence. Data were analysed using Pearson correlation, multiple regression analyses, and mediation analysis using the PROCESS macro.

**Results:**

Students demonstrated moderately high levels of debriefing experience, clinical decision-making, and competence. Simulation debriefing showed strong positive associations with both clinical decision-making and competence. Regression analyses identified debriefing quality and clinical rotation frequency as strong predictors. Mediation analysis revealed a significant partial mediation effect, indicating that debriefing improves competence partly by strengthening clinical decision-making processes.

**Conclusions:**

Simulation debriefing plays a pivotal role in enhancing students' cognitive integration of simulation experiences, supporting the development of informed clinical judgment and competence. Strengthening debriefing practices is therefore essential for advancing simulation-based nursing education and supporting readiness for clinical practice.

## Introduction

Simulation-based education has become an essential component of contemporary nursing education because it enables students to apply theoretical knowledge, practice clinical skills, and respond to realistic patient-care situations in a safe learning environment (1, 2). By allowing students to make clinical decisions without exposing real patients to harm, simulation helps bridge the gap between classroom learning and clinical practice (3). However, the educational value of simulation depends not only on the scenario itself but also on the quality of reflection and feedback that follows it. For this reason, simulation debriefing is widely recognized as a key element in transforming simulated experiences into meaningful clinical learning (4).

Debriefing provides nursing students with a structured opportunity to review their actions, analyze their clinical reasoning, discuss alternative interventions, and receive constructive feedback (5). Through this reflective process, students become more aware of how they interpret patient data, prioritize care needs, and choose appropriate nursing actions (6). Therefore, debriefing may play an important role in strengthening clinical decision-making, which is a central requirement for safe and competent nursing practice (7).

Clinical decision-making is a complex process that involves collecting and interpreting patient information, identifying priorities, selecting appropriate interventions, and evaluating patient outcomes (8). In nursing practice, decisions are often made under conditions of uncertainty, limited time, and changing patient needs. For nursing students, developing this ability is particularly important because clinical competence depends not only on knowledge and technical skills but also on the ability to apply them appropriately in real clinical situations (9). Thus, clinical decision-making can be understood as a mechanism through which learning experiences contribute to the development of clinical competence (10–12).

The proposed relationship between simulation debriefing, clinical decision-making, and clinical competence is supported by several theoretical perspectives. Kolb's Experiential Learning Theory explains learning as a cycle of concrete experience, reflection, conceptualization, and active experimentation (13). In simulation-based education, the simulation scenario provides concrete experience, while debriefing facilitates reflection and interpretation. Through guided reflection, students can examine the decisions they made, connect their actions with theoretical knowledge, and develop improved strategies for future practice. This process is expected to enhance clinical decision-making, which in turn supports the development of clinical competence (14, 15).

Cognitive Continuum Theory also provides a useful explanation for this pathway. The theory views clinical judgment as a movement between intuitive and analytical reasoning (16). During debriefing, students are encouraged to examine whether their decisions were based on intuition, evidence, previous knowledge, or incomplete reasoning. This helps them become more deliberate and accurate in their clinical judgments (17). Similarly, Feedback Intervention Theory emphasizes the importance of specific and constructive feedback in improving performance (18). In debriefing sessions, feedback allows students to recognize strengths, identify gaps, and refine their future decision-making processes (19).

Although previous studies have highlighted the benefits of simulation and debriefing in nursing education, the specific mechanism through which debriefing contributes to clinical competence remains insufficiently understood (20, 21). Much of the existing literature has focused on the direct benefits of simulation-based learning, such as improving confidence, clinical skills, or satisfaction. However, less attention has been given to whether clinical decision making explains how debriefing improves students' clinical competence. This gap is important because decision-making represents the cognitive process through which students translate reflection and feedback into competent clinical performance (14, 22).

In Egypt, simulation-based education has increasingly been integrated into nursing curricula to better prepare students for complex healthcare environments (23, 24). However, limited evidence is available regarding how simulation debriefing influences nursing students' clinical decision-making and competence within this context. Understanding this relationship may help nursing educators design more effective debriefing strategies that strengthen students' reasoning abilities and prepare them for safe, evidence-based clinical practice (25, 26).

Accordingly, this study aimed to examine the relationship between simulation debriefing, clinical decision-making, and clinical competence among nursing students. Specifically, the study tested whether clinical decision-making mediates the relationship between simulation debriefing and clinical competence (23, 26). Based on theoretical and empirical literature, the following hypotheses were proposed:

H1: Simulation debriefing is positively associated with nursing students' clinical competence.
H2: Simulation debriefing is positively associated with nursing students' clinical decision-making.
H3: Clinical decision-making is positively associated with nursing students' clinical competence.
H4: Clinical decision-making mediates the relationship between simulation debriefing and clinical competence among nursing students.

## Methods

### Research design and setting

This study employed a multi-center, cross-sectional descriptive design, adhering to the Strengthening the Reporting of Observational Studies in Epidemiology (STROBE) guidelines (50). Data were gathered from two nursing faculties—Mansoura University and Zagazig University—in Egypt. Both institutions function under the supervision of the Egyptian Ministry of Higher Education and Scientific Research and follow the nationally approved standards for nursing education. Each college comprises nine specialized academic departments covering diverse nursing disciplines.

### Sample size and study participants

To be eligible for participation in this study, students were required to be currently enrolled in the third or fourth year of a Bachelor of Science in Nursing (BSN) program at one of the participating universities. Participants must have had prior exposure to at least one simulation-based learning session that included a structured debriefing component. Students were included if they were willing to provide informed consent and could complete the questionnaire independently. Students were excluded if they had no prior experience with simulation-based training, were on academic leave during the data collection period, or were enrolled in bridging or postgraduate nursing programs, as their clinical exposure and educational experiences may differ significantly from those of regular undergraduate students.

The sample size was determined using G*Power 3.1.9.7 software (27). For the correlation, the bivariate normal test, which was determined using a power of.95, an alpha level of.05, and an effect size of 0.15, a minimum of 476 participants were required. 650 people were added to the sample size. A stratified random sampling technique was employed to ensure proportional representation from both participating universities and across academic years (third and fourth year). Within each stratum, students were randomly selected using student enrolment lists provided by the academic affairs departments. This approach minimized selection bias and ensured that the sample accurately reflected the diversity of nursing students' experiences with simulation-based education in both institutions, see Figure 1.

**Figure 1:**
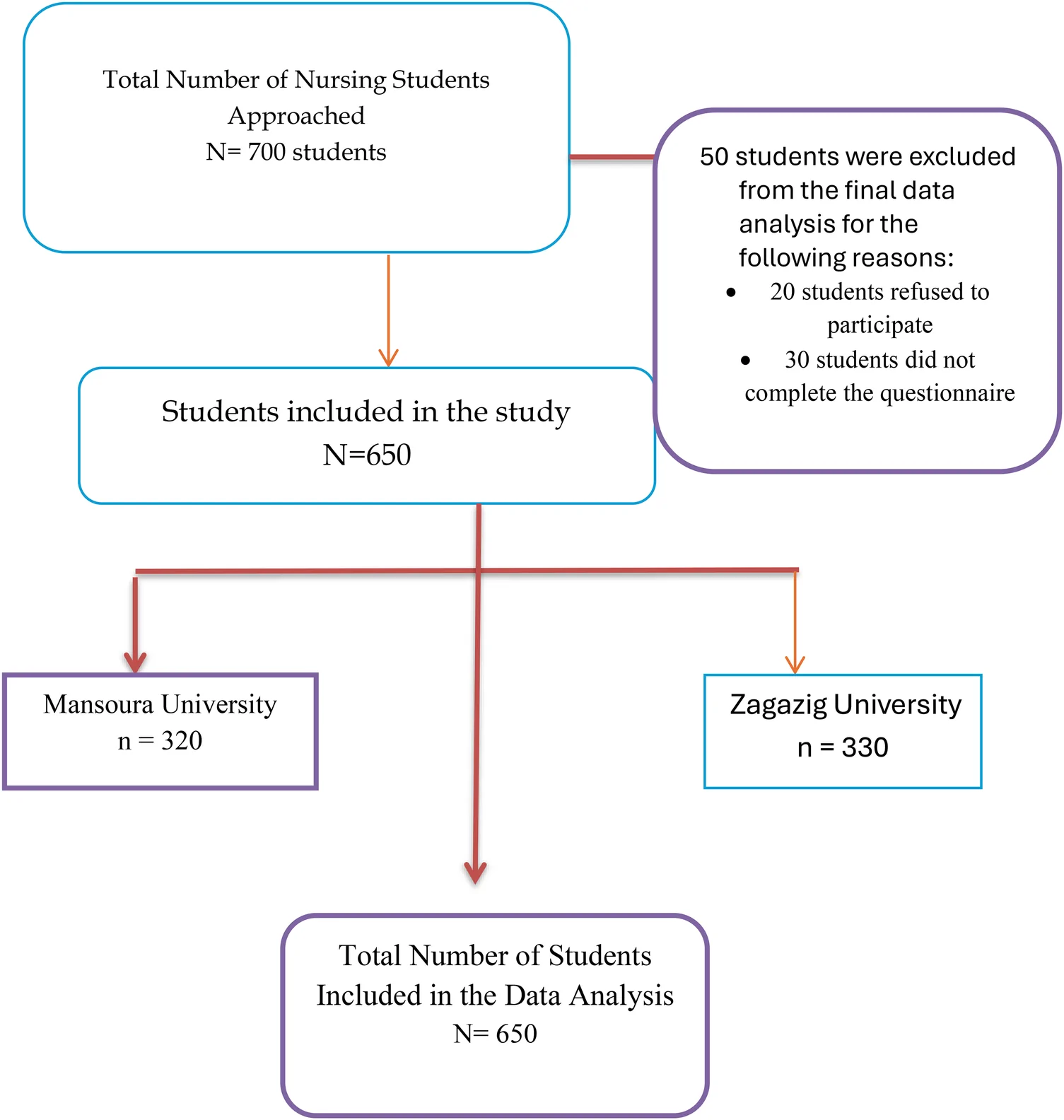
Flowchart of participant enrolment and allocation.

### Instruments

#### The demographic form

Participants completed a demographic data form designed to capture relevant personal, academic, and family background information. This included age, sex, occupation status, place of residence, type of family, and perceived family income. Parental educational levels were also collected to assess potential socio-educational influences. Academic information comprised the number of completed clinical rotations, previous participation in simulation sessions, and the types of debriefing experiences encountered.

#### The debriefing experience scale

The Debriefing Experience Scale (DES) is a self-administered instrument developed to assess nursing students' perceptions of the quality and effectiveness of simulation debriefing. The scale comprises 19 items organized into four domains: Learning and Making Connections (8 items), Analyzing Thoughts and Feelings (3 items), Facilitator Skill in Guiding Debriefing (5 items), and Appropriate Facilitator Input (3 items). Each item is rated on a five-point Likert scale ranging from 1 (strongly disagree) to 5 (strongly agree), with higher scores indicating more positive debriefing experiences.

Previous psychometric evidence was established by Xie et al. (28), who validated the simplified Chinese version of the DES and reported excellent internal consistency (Cronbach's *α* = 0.90 for the experience scale and.92 for the importance scale) and strong test–retest reliability (ICC = 0.86, 95% CI:0.64–0.98). The tool demonstrated excellent internal consistency in the current sample (Cronbach's *α* = 0.907).

#### A nursing competence assessment tool

The Nursing Competence Assessment Tool (NCAT), introduced by Huang et al. (29), evaluates undergraduate nursing students' competence across 30 items divided into six domains: Medical-Related Knowledge, Basic Nursing Skills, Communication and Cooperation, Lifelong Learning, Global Vision, and Critical Thinking (5 items each). Responses are captured on a five-point Likert scale from 1 (strongly disagree) to 5 (strongly agree), with higher aggregate scores indicating greater competence (29). Huang et al. reported robust psychometric performance: Subscale internal consistency was excellent (Cronbach's *α* = 0.85–0.92), and test–retest stability was strong (ICC = 0.83). In the present study, the scale maintained outstanding internal consistency (Cronbach's *α* = 0.912).

#### Nursing decision-making instrument-revised 2014 (NDMI-R 2014)

The Nurse Decision Making Instrument (NDMI) was originally developed by Lauri and Salanterä (2001) to evaluate whether nurses and nursing students rely more on analytic or intuitive approaches in their practice (25). Its initial form included 56 items across four sequential decision-making stages: gathering patient data, analyzing that information to identify nursing problems, planning and executing nursing interventions, and monitoring and evaluating both interventions and patient status. After factor analysis, Lauri and Salanterä reduced the scale to 24 items six per stage where odd-numbered items assess analytic decision-making in well-structured contexts and even-numbered items capture intuitive decision-making under uncertainty or time pressure (30). Phillips (2015) later refined this tool into the Nursing Decision-Making Instrument–Revised 2014 (NDMI-R 2014), retaining the same four domains: Data Collection (Items 1–6), Data Processing and Problem Identification (7–12), Action Planning (13–18), and Implementation, Monitoring, and Evaluation (19–23, 31). Each item is rated from 1 (never/almost never) to 5 (almost always/always), with higher total scores reflecting a greater propensity for intuitive decision-making. Reliability testing of the NDMI-R 2014 has shown strong internal consistency, with Cronbach's *α* coefficients of.85 for the analytic subscale and.83 for the intuitive subscale. Parker (2014) confirmed its construct and content validity through exploratory factor analysis (32). In the present study, psychometric evaluation revealed a Cronbach's *α* for the subscales Data Collection, Data Processing and Problem Identification, Action Planning, and Implementation, Monitoring, and Evaluation (19–23) were 0.807, 0.849, 0.932, and 0.664, respectively. In addition, the Cronbach's *α* for the analytic and intuitive were 0.697 and 0.907, respectively and 0.912 for the full 24-item instrument.

A pilot study was conducted with 60 nursing students to evaluate the clarity, feasibility, and psychometric soundness of the survey instruments. Participants in the pilot study were similar in characteristics to the main sample in terms of academic year and exposure to simulation-based learning. The pilot aimed to determine the average time required to complete the questionnaire, identify ambiguous or culturally sensitive items, and ensure smooth administration procedures. The average completion time was approximately 20 min, and minor linguistic adjustments were made to improve wording clarity without altering the original meaning of any items.

Internal consistency reliability was examined using Cronbach's alpha coefficients for each scale, which demonstrated strong reliability (*α* values ranging between 0.912 and 0.970 across the instruments). The findings indicated that no additional modifications were required, confirming the instruments' suitability for the primary study.

### Data collection

Data were collected over a two-month period, from April to May 2025, following the receipt of ethical approval from the participating universities. After coordination with the academic affairs departments, official lists of eligible third- and fourth-year nursing students were obtained to facilitate random selection. The researchers then contacted the selected students in coordination with course coordinators to schedule appropriate times for data collection. Before distributing the questionnaires, the researchers clearly explained the study's purpose, procedures, and ethical considerations, emphasizing that participation was voluntary and that responses would remain anonymous and confidential. Students who agreed to participate provided written informed consent prior to inclusion.

Data were gathered in controlled and distraction-free environments, such as quiet lecture halls and university libraries, during students' free periods to minimize academic disruption. Each session lasted approximately 15–20 min, during which participants completed the self-administered questionnaires independently under the supervision of the research team, ensuring clarity and completeness of responses.

### Ethical considerations

This study was approved by the Research Ethics Committee of the Faculty of Nursing at Zagazig University, Egypt (274). All procedures complied with relevant regulations, national legislation, and the ethical standards of the Declaration of Helsinki, with participant welfare as the foremost concern. Prior to participation, individuals received detailed explanations of the study's aims and were informed that involvement was entirely voluntary and responses would be anonymized. Confidentiality was assured, and collected data were accessible only to designated members of the research team. Written informed consent was obtained from every participant before data collection began.

### Data analysis

The Statistical Package for the Social Sciences (SPSS) version 26.0 was used to analyse the data. Participants' demographics and study data were summed up using descriptive statistics, such as mean, standard deviation (SD), frequency, and percentage. The Kolmogorov–Smirnov test was used to determine whether the data was normal, and it was discovered that all the variables were normally distributed. To investigate variations in clinical competence and decision-making among sociodemographic categories, one-way ANOVA and independent samples t-tests were used.

Tukey's *post hoc* tests were used to determine group differences based on significant ANOVA results. Pearson's correlation coefficient (r) was used to evaluate the connections between clinical decision-making, competency, and simulation debriefing. Multiple linear regression analyses were performed to find predictors of clinical decision-making and competence, and *β* coefficients, 95% CIs, and *p*-values were reported. Multiple linear regression analyses were performed to identify factors associated with clinical decision-making and nursing competence. The dependent variables were clinical decision-making and nursing competence, while the independent variables included simulation debriefing and the sociodemographic characteristics (age, gender, residence, family income, marital status, family type, number of completed clinical rotations, and participation in simulation sessions). All variables were entered simultaneously using the Enter method. Before model estimation, the assumptions of multiple linear regression were evaluated.

Multicollinearity was assessed using the Variance Inflation Factor (VIF) and tolerance statistics. For the competence model, VIF values ranged from 1.125 to 2.063, with tolerance values ranging from 0.485 to 0.889. For the clinical decision-making model, VIF values ranged from 1.047 to 2.059, with tolerance values ranging from 0.486 to 0.955, indicating no evidence of problematic multicollinearity. In addition, linearity, normality of residuals, homoscedasticity, and independence of errors were examined and confirmed before interpreting the regression results. Regression coefficients are presented as unstandardized coefficients (B) and standardized coefficients (*β*), 95% CIs, with their corresponding 95% confidence intervals and *p*-values.

In order to investigate if clinical decision-making mediated the association between competency and simulation debriefing, mediation analysis was also conducted. With 95% bias-corrected confidence intervals, bootstrapping techniques (5,000 samples) were used to estimate the indirect, direct, and total impacts. For all analyses, a *p*-value of less than 0.05 was deemed statistically significant.

## Results

Table 1 presents the descriptive statistics of the main study variables among nursing students (*N* = 650). The mean score for simulation debriefing was 69.16 ± 20.35, clinical decision-making was 88.18 ± 21.22, and competence was 111.81 ± 26.41.

**Table 1:** Descriptive statistics of study variables (*N* = 650).

Variables	Minimum	Maximum	Mean ± SD
Simulation Debriefing	19.00	95.00	69.16 ± 20.35
Clinical Decision-Making	48.00	120.00	88.18 ± 21.22
Competence	68.00	150.00	111.81 ± 26.41

Table 2 shows the association between participants' sociodemographic characteristics and their levels of clinical decision-making and competence. Age was significantly associated with both clinical decision-making (F = 14.89, *p* < 0.001) and competence (F = 5.97, *p* = 0.003), with 21-year-old students scoring higher than those aged 20 or 22 years.

**Table 2:** Relationship between sociodemographic characteristics of participants and the levels of clinical decision-making and competence (*N* = 650).

Variables	Categories	*n*	%	Clinical Decision-Making		Competence	
				Mean ± SD	t/f (*p*-value)	Mean ± SD	t/f (*p*-value)
Age							
	20.00^a^	327	50.3	84.88 ± 20.44	14.89 (<.001) *b > a, b > c	112.72 ± 27.19	5.97 (0.003) *a > c, b > c
	21.00^b^	208	32.0	94.64 ± 22.40		114.52 ± 23.60	
	22.00^c^	115	17.7	85.88 ± 18.57		104.34 ± 27.83	
Gender							
	Male	260	40.0	85.80 ± 19.12	−2.34 (.019)	109.85 ± 24.48	−1.54 (0.122)
	Female	390	60.0	89.77 ± 22.40		113.12 ± 27.58	
Residence							
	Rural	402	61.8	86.50 ± 20.89	−2.58 (.010)	114.52 ± 26.11	3.35(<0.001)
	Urban	248	38.2	90.91 ± 21.51		107.42 ± 26.36	
Income							
	Not enough	121	18.6	83.26 ± 19.31	−2.84 (.005)	108.04 ± 31.43	−1.74 (0.081)
	Enough	529	81.4	89.31 ± 21.49		112.68 ± 25.08	
Marital status							
	Single	572	88.0	88.47 ± 22.06	.95 (.339)	112.86 ± 26.54	2.75 (0.006)
	Married	78	12.0	86.02 ± 13.45		104.14 ± 24.32	
Family type							
	Nuclear	304	46.8	89.50 ± 24.41	1.49 (.137)	116.59 ± 27.32	4.38 (<0.001)
	Extended	346	53.2	87.02 ± 17.91		107.61 ± 24.89	
Number of Completed Clinical Rotations							
	1.00^a^	207	31.8	76.55 ± 20.62	96.580 (<.001) *c > a, d > a, e > a, d > b, e > b, e > c, e > d	89.91 ± 21.56	97.94 (<0.001) *b > a, c > a, d > a, e > a, c > b, d > b, e > b
	2.00^b^	120	18.5	79.88 ± 8.67		109.19 ± 17.22	
	3.00^c^	152	23.4	88.66 ± 20.32		113.08 ± 27.14	
	4.00^d^	84	12.9	93.09 ± 20.44		131.32 ± 24.26	
	≥5.00^e^	87	13.4	114.03 ± 9.13		132.00 ± 12.12	
Participation in Simulation Sessions							
	1.00^a^	261	40.2	91.36 ± 23.00	3.539 (.007) *a > d,	113.69 ± 21.99	5.81 (<0.001) *a > e, b > c, b > e, d > e
	2.00^b^	89	13.7	89.06 ± 17.24		118.48 ± 18.77	
	3.00^c^	96	14.8	87.45 ± 15.07		107.76 ± 31.08	
	4.00^d^	160	24.6	84.35 ± 23.26		111.48 ± 30.85	
	≥5.00^e^	44	6.8	83.04 ± 18.51		97.27 ± 29.44	

*a, b, c, d, and e indicate the group labels compared in Tukey's *post hoc* test.

Female students demonstrated significantly higher clinical decision-making scores than males (t = −2.34, *p* = 0.019). Participants from urban areas scored significantly higher in clinical decision-making (t = −2.58, *p* = 0.010), whereas those from rural areas showed higher competence (t = 3.35, *p* < 0.001). Students with sufficient income had significantly better clinical decision-making skills than those with insufficient income (t = −2.84, *p* = 0.005). Marital status was significantly related to competence (t = 2.75, *p* = 0.006), with single students scoring higher than married ones.

Family type also showed a significant association with competence (t = 4.38, *p* < 0.001), as students from nuclear families demonstrated higher competence levels. The number of completed clinical rotations was strongly associated with both clinical decision-making (F = 113.91, *p* < 0.001) and competence (F = 97.94, *p* < 0.001), with students who had completed five or more rotations achieving the highest scores. A statistically significant difference in nursing competence was observed according to the number of simulation sessions attended (*p* < 0.001). Students who participated in ≥5 simulation sessions had the lowest mean competence score (97.27 ± 29.44), whereas those who participated in two simulation sessions demonstrated one of the highest competence scores (118.48 ± 18.77). *Post hoc* analysis showed that the ≥5-session group had significantly lower competence than the one-session, two-session, and four-session groups, indicating a non-monotonic association between simulation-session frequency and competence.

Table 3 demonstrates significant positive correlations among all study variables. Simulation debriefing showed a strong positive correlation with clinical decision-making (*r* = 0.747, *p* < 0.001) and competence (*r* = 0.694, *p* < 0.001). Similarly, clinical decision-making was strongly correlated with competence (*r* = 0.720, *p* < 0.001). These findings indicate that higher levels of simulation debriefing are associated with improved clinical decision-making and competence among nursing students.

**Table 3:** Correlation between study variables (*N* = 650).

Variables		Simulation Debriefing	Clinical Decision-Making	Competence
Simulation Debriefing	R	1		
	P			
Clinical Decision-Making	R	0.747^a^	1	
	P	<0.001		
Competence	R	0.694^a^	0.720^a^	1
	P	<0.001	<0.001	

a Correlation is significant at the 0.01 level (2-tailed).

The multiple linear regression model for clinical decision-making was statistically significant (R² = 0.414, Adjusted R² = 0.403, *p* < 0.001). Age (*β* = 0.078, *p* = 0.001), gender (female; *β* = 0.091, *p* = 0.020), income (enough; *β* = 0.074, *p* = 0.004), and the number of completed clinical rotations were significant predictors. Students who completed 2, 3, 4, and ≥5 clinical rotations had progressively higher clinical decision-making scores (*β* = 0.119, 0.294, 0.331, and 0.705, respectively; all *p* < 0.05).

The regression model for competence was also significant (R² = 0.464, Adjusted R² = 0.453, *p* < 0.001). Competence was significantly associated with residence (urban; *β* = −0.137, *p* < 0.001), family type (nuclear; *β* = −0.193, *p* < 0.001), and the number of completed clinical rotations. Students with 2, 3, 4, and ≥5 clinical rotations had markedly higher competence scores (*β* = 0.367, 0.440, 0.650, and 0.614, respectively; all *p* < 0.001), Table 4.

**Table 4:** Multiple linear regression of factors associated with clinical decision-making and competence among nursing students.

Variables	Categories	Clinical Decision-Making				Competence			
		B	Β	95% CI for B	*P*-value	B	Β	95% CI for B	*P*-value
Age		2.181	0.078	0.430–3.933	0.015	−1.445	−0.041	−3.74-0.85	0.217
Gender	Male	Reference							
	Female	3.959	0.091	1.321–6.597	0.003				
Residence	Rural	Reference				Reference			
	Urban	1..286	0.029	−1.803–4.376	0.414	−7.472	−0.137	−11.06–3.88	<0.001
Income	Not enough	Reference							
	Enough	4.056	0.074	0.539–7.573	0.024				
Marital status	Single					Reference			
	Married					−3.178	−0.039	−8.62–2.28	0.254
Family type	Nuclear					Reference			
	Extended					−10.238	−0.193	−13.43–7.04	<0.001
Number of Completed Clinical Rotations	1.00	Reference				Reference			
	2.00	5.969	0.119	1.923–10.014	0.004	22.907	0.367	18.03–27.78	<0.001
	3.00	16.901	0.294	11.996–21.806	<0.001	31.458	0.440	25.60–37.30	<0.001
	4.00	19.169	0.331	14.264–24.073	<0.001	46.811	0.650	41.10–52.51	<0.001
	≥5.00	40.146	0.705	35.833–44.460	<0.001	43.560	0.614	38.37–48.74	<0.001
Participation in Simulation Sessions	1.00	Reference				Reference			
	2.00	−5.759	−0.093	−10.545–−0.972	0.018	−4.218	−0.055	−9.84−1.41	0.142
	3.00	−0.056	−0.001	−4.614–4.501	0.981	−12.005	−0.161	−17.54–6.46	<0.001
	4.00	−1.008	−0.020	−5.086–3.071	0.628	−6.451	−0.105	−11.30–1.59	0.009
	≥5.00	7.938	0.094	2.088–13.788	0.008	6.395	0.061	-0.49−13.28	0.069

*R2 = 0.414, Adjusted R2 = 0.403, *p* < 0.001, *R2 = 0.464, Adjusted R2 = 0.453, *p* < 0.001. B, Unstandardized coefficient; β, Standardized coefficient; CI, Confidence Interval.

The model was built using the PROCESS SPSS macro with competence as the dependent variable, clinical decision-making as the mediating variable, and simulation debriefing as the independent variable. Figure 2 illustrates this model. Mediation analysis revealed that simulation debriefing had both direct and indirect effects on nursing students' competence. Table 5 presents the direct, indirect, and total association of simulation debriefing and competence, with clinical decision-making as a mediator. The mediator variable (MV) model indicated that simulation debriefing had a strong and statistically significant positive association with clinical decision-making (*β* = 0.609, *p* < 0.001), explaining 61.2% of its variance (R² = 0.612, *p* < 0.001). In the dependent variable (DV) model, both clinical decision-making (*β* = 0.370, *p* < 0.001) and simulation debriefing (*β* = 0.340, *p* < 0.001) were significant predictors of competence, with the model accounting for 69.9% of the variance (R² = 0.699, *p* < 0.001).

**Figure 2:**
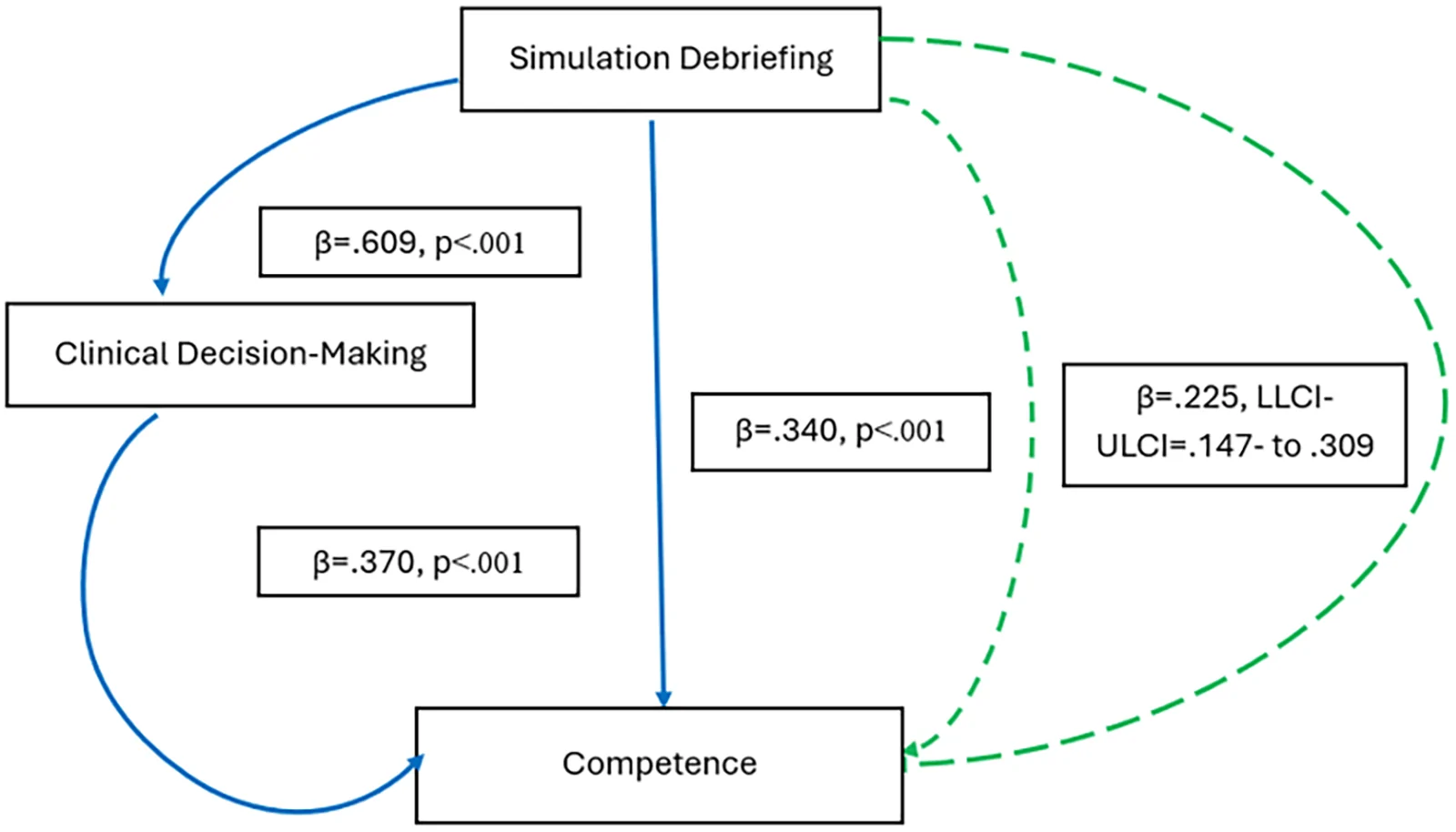
Statistical mediation model linking simulation debriefing, clinical decision-making, and nursing competence.

**Table 5:** Direct, indirect, and total associations between simulation debriefing and competence through clinical decision-making.

Models	B	β	SE	T	95% CI	*P*-value
MV Model						
Simulation Debriefing → Clinical Decision-Making	0.635	00.609	0.031	20.478	0.574–0.695	<0.001
Model Summary R2=.612, F = 144.573, *p* < 0.001						
DV Model						
Clinical Decision-Making → Competence	0.460	0.370	0.043	10.619	0.375–0.545	<0.001
Simulation Debriefing → Competence	0.441	0.340	0.044	10.091	0.355–0.527	<0.001
Model Summary R2=.699, F = 185.672, *p* < 0.001						
Total Effect						
Simulation Debriefing → Competence	0.733	0.565	0.037	19.894	0.661–0.806	<0.001
Age (Covariate)	−3.949	−0.113	0.925	−4.269	−5.765–−2.133	<0.001
Residence (Covariate)	−13.164	−0.242	1.313	−10.024	−15.743–−10.585	<0.001
Marital status (Covariate)	1.676	0.021	2.157	.777	−2.559–5.912	0.437
Family type (Covariate)	−10.420		1.275	−8.175	−12.923–−7.917	<0.001
Number of Completed Clinical Rotations (Covariate)	5.488	0.300	0.531	10.337	4.445 to 6.530	<.001
Participation in Simulation Sessions (Covariate)	−0.391	−0.021	0.465	−0.842	−1.303–0.521	0.400
Model Summary R2=.646, F = 167.015, *p* < 0.001						
Indirect effect	β			BootSE	BootLLCI	BootULCI
Simulation Debriefing → Clinical Decision-Making → Competence	0.225			0.041	0.147	0.309

B, unstandardized coefficient; β, standardized coefficient; SE, standard error; t, t-value; CI, confidence interval; LLCI, lower limit of confidence interval; ULCI, upper limit of confidence interval; p, probability value; R², coefficient of determination; F, F-statistic; MV, mediator variable model; DV, dependent variable model; BootSE, bootstrap standard error.

The total effect model demonstrated that simulation debriefing had a significant positive association with competence (*β* = 0.565, *p* < 0.001). Among the covariates, age (*β* = −0.113, *p* < 0.001), residence (*β* = −0.242, *p* < 0.001), family type (*p* < 0.001), and number of completed clinical rotations (*β* = 0.300, *p* < 0.001) were significant predictors of competence, whereas marital status and participation in simulation sessions were not statistically significant (*p* > 0.05). The overall model explained 64.6% of the variance in competence (R² = 0.646, *p* < 0.001).

Furthermore, the indirect association of simulation debriefing with competence through clinical decision-making was statistically significant [*β* = 0.225, BootSE = 0.041, 95% CI (0.147, 0.309)], indicating partial mediation.

## Discussion

Simulation debriefing is widely recognized as a cornerstone of effective nursing education, providing students with opportunities to reflect, analyze, and integrate their simulation experiences into clinical reasoning and practice [(5, 8, 20, 51, 52). In the current cross-sectional study, simulation debriefing was positively associated with nursing students' clinical decision-making and clinical competence. Therefore, the findings should be interpreted as evidence of significant relationships among these variables rather than as evidence of causal effects.

The significant positive correlations found between simulation debriefing, decision-making, and competence are in line with the pedagogical foundations advocated by Kolb's experiential learning theory. Specifically, guided reflective debriefing may support the “reflective observation” phase, which promotes the transformation of concrete simulation experiences into deeper clinical reasoning and learning (20, 33). From this perspective, students who report more effective debriefing experiences may also demonstrate stronger clinical decision-making and competence because reflection helps them review actions, reconsider clinical cues, and connect simulation experiences with prior knowledge. This mechanism of reflection and integration is essential for developing sound clinical judgment and may help explain the associations observed in the present study, which are also supported by current literature (11, 34, 35).

Furthermore, the study's findings reinforce the importance of simulation-based activities as a bridge between theoretical knowledge and practical application in nursing education. Such activities, through structured debriefing, are associated with students' ability to consolidate skills in a safe learning environment, build teamwork, and strengthen confidence in clinical learning (36). Consistent with the theoretical framework presented in the Introduction, this interpretation is best understood through Kolb's experiential learning theory, Cognitive continuum theory, and feedback intervention theory. Together, these perspectives suggest that debriefing provides students with opportunities to reflect on their experiences, examine intuitive and analytical reasoning, and receive feedback that may be linked to better decision-making and competence (13, 16).

Consistent with the current findings, recent studies have reported that students who engage in guided reflective debriefing sessions exhibit improved decision-making and problem-solving abilities (37). Additionally, a comprehensive review indicated that effective simulation activities significantly advance communication skills, clinical reasoning, teamwork, and the ability to manage complex scenarios (38, 39). Tamilselvan et al. (2023) observed in their review that a significant finding was the provision of opportunities for male undergraduate nursing students to engage in “gender-related nursing care,” including maternal care. This encompasses the application of skills that are not typically authorized for the care of female patients within certain cultural and national contexts (40).

Age emerged as a significant factor, with 21-year-old students exhibiting greater clinical decision-making skills and competence. Given the cross-sectional nature of the study, this finding indicates an association between age and students' reported decision-making and competence scores, but it does not establish that age directly improves these outcomes (41). Empirical evidence also indicates that clinical decision-making abilities may vary with age and academic progression among nursing students, reflecting the possible role of developmental readiness and increased exposure to diverse clinical contexts in higher levels of competence.

Similarly, the observed gender differences, favoring female students in clinical decision-making, should be interpreted as group differences within this sample rather than as evidence that gender causes better decision-making. Several authors suggest that enhanced empathy and communication, traits often associated with female nursing students, may be related to more effective patient-centered decision-making (42).Additionally, Ogunmuyiwa et al. (2025) found that gender significantly influences competencies (*p* < 0.05), with female nursing students demonstrating higher clinical competence. In contrast, age does not have a significant impact on nursing students' clinical competence (43).

Students' socioeconomic and environmental backgrounds were also associated with learning outcomes. Those with adequate income and residing in urban settings scored higher in clinical decision-making, which may reflect greater access to educational, technological, or simulation-related resources. However, this interpretation remains tentative because the study design does not allow causal conclusions. Conversely, students from rural areas demonstrated higher competence, a finding that may be related to contextual adaptability, prior practical exposure, or other unmeasured educational experiences, a trend observed in recent cross-cultural studies (8, 44). Moreover, the findings that single students and those from nuclear families reported higher competence might reflect fewer familial constraints and greater academic focus, aligning with earlier nursing education studies examining psychosocial influences on learning outcomes, for example the study of Wang et al. (2025), who documented the complex interplay between metacognitive processes, situational factors, and individual differences in the development of clinical decision-making skills among nursing students (45).

The study also showed that clinical exposure and experiential learning were positively correlated with competence. This aligns with Alsadi et al. (2025), who found that nursing students consider “support” during simulation-based activities, clear “objectives and information,” and high “fidelity” as key aspects of effective simulation design (46). An “active learning” approach was viewed as the most vital educational practice. Consistent with Kolb's experiential learning theory, repeated clinical rotations and simulation sessions may be associated with stronger decision-making and procedural abilities because they provide students with opportunities for experience, reflection, and application (13).

The mediation analysis revealed that clinical decision-making partially mediated the relationship between simulation debriefing and competence. Because the data were cross-sectional, this mediation finding should be interpreted as evidence of an indirect statistical association rather than proof of a causal pathway. This finding is consistent with theoretical models emphasizing that debriefing may be linked to both forward reasoning, such as cue acquisition, and backward reasoning, such as cue interpretation and hypothesis generation (47). Within the framework used in this study, Hammond's cognitive continuum theory helps explain how students may move between intuitive and analytical reasoning during and after simulation, while feedback intervention theory explains how structured feedback during debriefing may be associated with refinement of decision-making processes (15, 48).

In line with Abdulmohdi and McVicar (2023), who noted a significant moderate rise in the “deduction” and “analysis” sub-scale scores along with the overall test score, indicating enhanced analytical decision-making skills through simulation, the data from think-aloud and observation methods show that students mainly used “forward” reasoning during the simulated patient's deterioration, primarily focusing on cue collection (5). Conversely, backward reasoning, emphasizing cue interpretation, was most evident in the debriefing data and aligned with survey results. Correct interpretation of critical cues proved more beneficial than merely counting the total cues in solving the main clinical case (49). Taken together, these findings suggest that effective debriefing is associated with better clinical decision-making, and that decision-making may serve as an important explanatory link between debriefing and clinical competence. However, longitudinal or experimental studies are needed to confirm the direction and causality of these relationships.

### Implications of the study

The findings clearly show that high-quality simulation debriefing significantly enhances nursing students' clinical decision-making and overall competence, with clinical decision-making acting as a key mechanism in this process. These insights provide strong justification for nursing programs to integrate structured, theory-based debriefing approaches across all simulation activities. Educators should adopt standardized debriefing models such as Debriefing for Meaningful Learning and Plus-Delta to ensure consistency, depth of reflection, and guided feedback. Additionally, programs should train simulation facilitators through targeted professional development in debriefing communication, cueing strategies, and learner-centered reflection. Embedding regular debriefing competency evaluation for educators will further guarantee high-quality learning experiences.

At an institutional level, nursing faculties should expand simulation opportunities and link them directly to clinical rotations to reinforce learning transfer. The strong effect of clinical rotations on both competence and decision-making highlights the need to schedule simulation and debriefing sessions progressively and align them with specific clinical objectives. Policies should prioritize early and repeated exposure to simulation debriefing, ensure adequate access to high-fidelity simulation resources, and integrate student-centered reflective activities into curricula. Administrators should also consider addressing sociodemographic differences identified in the study by providing additional academic support for students from lower-income or rural backgrounds, ensuring equitable access to competence-building activities.

### Limitations and future directions

This study is limited by its cross-sectional design, which restricts causal interpretation of the relationships among debriefing, clinical decision-making, and competence. Data were collected through self-report instruments, which may introduce social desirability or recall bias. Although the sample is sizable and drawn from two major universities, the findings may not fully represent all nursing institutions in XX or other contexts. Future research should employ longitudinal or experimental designs to track changes in competence and decision-making over time and evaluate the sustained impact of structured debriefing. Furthermore, integrating objective performance measures—such as OSCE scores, simulation performance analytics, or clinical preceptor evaluations—would strengthen understanding of how debriefing translates into real clinical performance.

## Conclusion

This study suggests that simulation debriefing is positively associated with nursing students' self-reported clinical decision-making and clinical competence. Debriefing may provide structured opportunities for reflection, feedback, and discussion of clinical actions, which can help students better understand their decision-making processes. Strengthening debriefing practices, therefore, may support nursing students' clinical learning and contribute to the development of decision-making and competence within simulation-based education. Future longitudinal or experimental studies are needed to confirm the direction and causal nature of these relationships.

## Data Availability

The datasets and materials of the current study are available from the corresponding author upon reasonable request.
